# Small-scale entrainment in inclined gravity currents

**DOI:** 10.1007/s10652-017-9514-3

**Published:** 2017-03-07

**Authors:** Maarten van Reeuwijk, Dominik Krug, Markus Holzner

**Affiliations:** 1 Department of Civil and Environmental Engineering, Imperial College London, London, SW7 2AZ UK; 2 Department of Mechanical Engineering, University of Melbourne, Victoria, 3010 Australia; 3 Institute of Environmental Engineering, ETH Zürich, 8039 Zurich, Switzerland

**Keywords:** Gravity current, Turbulent entrainment, Small-scale turbulence

## Abstract

We investigate the effect of buoyancy on the small-scale aspects of turbulent entrainment by performing direct numerical simulation of a gravity current and a wall jet. In both flows, we detect the turbulent/nonturbulent interface separating turbulent from irrotational ambient flow regions using a range of enstrophy iso-levels spanning many orders of magnitude. Conform to expectation, the relative enstrophy isosurface velocity $v_n$ in the viscous superlayer scales with the Kolmogorov velocity for both flow cases. We connect the integral entrainment coefficient *E* to the small-scale entrainment and observe excellent agreement between the two estimates throughout the viscous superlayer. The contribution of baroclinic torque to $v_n$ is negligible, and we show that the primary reason for reduced entrainment in the gravity current as compared to the wall-jet are 1) the reduction of $v_n$ relative to the integral velocity scale $u_T$; and 2) the reduction in the surface area of the isosurfaces.

## Introduction

Density currents are significant in a variety of natural phenomena, ranging from cold water overflows in the ocean to thunderstorm outflows or sea-breeze fronts in the atmosphere. They are of importance to many engineering applications, such as water quality management in reservoirs where dense inflows may carry suspended matter and dissolved solids and dominate the dispersion of pollutants [1]. A central aspect that controls the dynamics of density currents is the entrainment of surrounding fluid into the turbulent flow. The entrainment in an inclined dense gravity current was first studied by Ellison and Turner [2] through laboratory experiments. In a first set of experiments, fluid that is lighter than its surroundings was emitted by a source under a sloping roof and in a second set of experiments a heavier fluid was emitted from a source on a sloping floor (the former being the ‘flipped’ configuration of the latter but otherwise producing identical flow and entrainment characteristics) [2]. It was found that the entrainment is proportional to the mean velocity of the turbulent layer *U* multiplied by an empirical relation *E*(*Ri*) of the Richardson number, which is the ratio between the stabilizing buoyancy force and destabilizing shear force [2]. Many authors have since tried to validate and extend the determination of *E* either empirically or based on theoretical modelling (e.g. [2–8]; see also [9] and references therein). However, there is still significant uncertainty in the accurate quantification and correct parametrization of *E* [5].

Overall, previous literature results are dominated by rather crude bulk measurements, which reveal general trends but allow limited access to local flow physics of entrainment. Indeed, one of the reasons for the still incomplete understanding of entrainment and quantification of *E* is the lack of understanding of the entrainment at small scales, i.e. locally occurring at the interface between turbulent and surrounding flow. It has been demonstrated recently that *E* can be understood from small-scale processes [6, 10, 11] since the global entrainment comes about through small scale viscous diffusion of vorticity that is augmented by the strongly convoluted interface separating turbulent from surrounding flow regions. However, our advancements in the understanding of the turbulent/nonturbulent interface (TNTI) have been mostly limited to flows without density contrast [12].

It has been shown in [10] that a local entrainment velocity $v_n$ can be defined as the propagation speed of the TNTI relative to the fluid. The properties of $v_n$ have been studied experimentally in a flow without mean shear [10], a round jet [13], and numerically in a temporal plane jet [11] shedding light on local physics of entrainment and their relationship with global entrainment. Van Reeuwijk and Holzner [11] systematically studied the TNTI over a broad range of threshold levels used to identify the interface. This approach was recently used to study the TNTI in penetrative convection [14], where it was found that the baroclinic torque term plays a surprisingly small role in the propagation of the TNTI.

In a recent experiment, Krug et al. [6, 15] carried out simultaneous three-dimensional recordings of both velocity and density to investigate small-scale properties of entrainment in an inclined gravity current. A main result was that $v_n$ was dominated by viscous diffusion, similar to results in non-stratified flows, while the influence of the baroclinic torque was found to be small [6]. Up to now, no numerical study focusing on small-scale entrainment on density currents has been carried out. The aim of the present study is to (1) carry out gravity current simulations and analyse the TNTI using the approach of van Reeuwijk and Holzner [11] and (2) study the influence of stratification on small-scale entrainment through comparison between wall jet and inclined gravity current. The flows are selected such that they have the same initial conditions, but the wall jet does not experience buoyancy effects whilst the gravity current does, allowing for a systematic investigation of the influence of stratification on the TNTI.

## Simulation details

**Fig. 1:**
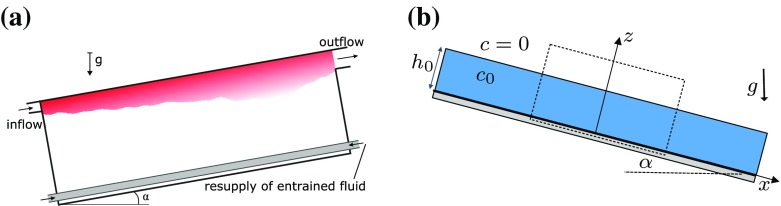
Definition sketches. **a** Experimental set-up of Krug et al. [19]. **b** Simulation set-up used in this paper

The wall jet and gravity current simulations we consider in this work are inspired on the experiment documented in Krug et al. [15] which in turn were based on the classical work of Ellison and Turner [2]. In the experiment, fresh water is injected into an inclined channel filled with a saline solution (see Fig. 1a) as a wall jet. Once inside the channel, the jet rapidly becomes a gravity current, and by gently resupplying the saline solution that is entrained into the inclined gravity current, a steady state situation is created.

The simulations comprise a temporal version of this experiment, namely the evolution of a negatively buoyant fluid layer as it flows down a slope of angle $\alpha$, as sketched in Fig. 1b. The flow physics are unaffected by flipping the problem upside-down as we consider a Boussinesq fluid. At time $t=0$, the layer has depth $h_0$, a uniform velocity *U*, and initial concentration $c_0$. The initial concentration is zero for $z>h_0$.

For the wall jet *c* is a passive scalar, whilst for the gravity current *c* is an active scalar (e.g. salinity). For both simulations, the angle is taken to be $\alpha =10^{\circ }$. The fluid inside the gravity current is heavier than the fresh water ($c=0$) in the ambient, causing the fluid to accelerate in the positive *x*-direction, transition to turbulence and flow down the slope as an inclined gravity current. In the case of the wall jet, the flow will transition to turbulence and decelerate because of the absence of buoyancy forcing.

Because of the problem set-up, the flow will remain homogeneous over the *x* and *y* direction, and its statistics will thus only depend on the vertical coordinate *z* and time *t* [16]. The temporal gravity current will therefore neither have a head nor a tail. Instead it can be thought to represent the body of the gravity current or a section of an ocean overflow (albeit in an idealised manner as there is no spatial development of the flow).

The simulations are carried out with SPARKLE, a code for direct numerical simulation (DNS) which numerically integrates the incompressible Navier-Stokes equations in the Boussinesq approximation

1 $$\begin{aligned} \frac{\partial \varvec{u} }{\partial t} + \varvec{u} \cdot \nabla \varvec{u}&= - \rho _{0}^{-1}\nabla p + \nu \nabla ^2 \varvec{u}+ \varvec{b}, \end{aligned}$$

2 $$\begin{aligned} \frac{\partial c}{\partial t} + \varvec{u} \cdot \nabla c&= D \nabla ^2 c, \end{aligned}$$

3 $$\begin{aligned} \nabla \cdot \varvec{u}&= 0. \end{aligned}$$

Here $\varvec{x} = (x, y, z)^T$ where *x*, *y* and *z* are the streamwise, lateral and vertical coordinate, $\varvec{u} = (u, v, w)^T$ is the fluid velocity, *p* is the (modified) pressure, $\varvec{b}=\beta \varvec{g} c$ is the buoyancy where $\varvec{g}=(\sin \alpha ,0,-\cos \alpha )g$ and $\beta =\rho _0^{-1} \partial \rho / \partial c |_{c_0}$, and $\nu$, *D* are the kinematic viscosity and molecular diffusivity, respectively.

The DNS code solves Eqs. (1–3) on a cuboidal domain and is fully parallelised making use of domain decomposition in two directions. The spatial differential operators are discretised using second order symmetry-preserving central differences [17], and time-integration is carried out with an adaptive second order Adams–Bashforth method [18]. Periodic boundary conditions are applied for the lateral directions. At the bottom wall, no-slip conditions are applied for the velocity and a Neumann (no-flux) boundary conditions for buoyancy. At the top, free-slip boundary conditions are applied for velocity and Neumann (no-flux) boundary for buoyancy.

In this paper, we will compare the entrainment in a temporal wall jet (WJ; $\beta =0)$ to the entrainment in a temporal gravity current (GC; $\beta > 0)$. Using the initial values *U*, $h_0$ and $c_0$, a Reynolds number $\hbox {Re}_0$ and Richardson number $\hbox {Ri}_0$ can be defined as

4 $$\begin{aligned} \hbox {Re}_0 = \frac{U h_0}{\nu }, \quad \hbox {Ri}_0 = \frac{B_0 \cos \alpha }{U^2}, \end{aligned}$$

where $B_0 = \beta g c_0 h_0$ is the integral buoyancy which is a conserved quantity in the simulations, as can be verified by integrating (2) over *z* and applying the boundary conditions.

Consistent with the experiment, $\hbox {Re}_0 = 3700$, for both the WJ and GC simulations; the inflow Richardson numbers $\hbox {Ri}_0 = 0$ and 0.11 for WJ and GC, respectively. The simulations are carried out on a large domain of $20\,h_0 \times 20\,h_0 \times 10\,h_0$ to ensure reliable statistics for this transient problem. A resolution of $N_x \times N_y \times N_z = 1536^2 \times 1152$, sufficient for DNS, is employed for both simulations. Further simulation details can be found in Table 1.

**Table 1:** Simulation data. The Taylor Reynolds number ${Re}_{\lambda }= \lambda u^{\prime }_T/\nu$, with $\lambda = \sqrt{15 \nu /\varepsilon _T }u^{\prime }_T$, $u^{\prime }_T= \sqrt{2e_T/3}$, where $\varepsilon _T=h^{-1} \int _0^{\infty }\varepsilon d z$ and $e_T=h^{-1}\int _0^{\infty }e \hbox{d} z$ are the top-hat values of the rate of turbulent dissipation and turbulent kinetic energy, respectively. $\Delta z$ is the grid resolution in the wall-normal (*z*-) direction and $\eta = (\nu ^3/\varepsilon _T)^{1/4}$ the Kolmogorov length scale. The resolution in streamwise and spanwise directions are $\Delta x =\Delta y =1.5 \Delta z$, respectively

Sim.	$N_x N_y N_z$	$L_x L_y L_z / h_0^3$	$\hbox {Re}_0$	$\hbox {Re}_{\lambda }$	$\Delta z/\eta$	$\hbox {Ri}_0$	$t_{run}/t^{\star }$	*E*
WJ	$1536^2 \times 1152$	$20^2 \times 10$	3700	135	0.48	0	100	0.09–0.10
GC	$1536^2 \times 1152$	$20^2 \times 10$	3700	118	0.78	0.11	300	0.02

*WJ* wall-jet simulation, *GC* gravity current simulation

## Self-similarity

Consistent with [2, 15] we define the characteristic thickness *h*, velocity $u_T$ and scalar concentration $c_T$ according to

5 $$\begin{aligned} u_T h = \int _0^\infty \overline{u} d z, \quad u_T^2 h = \int _0^\infty \overline{u}^2 d z, \quad c_T h = \int _0^\infty \overline{c} d z. \end{aligned}$$

**Fig. 2:**
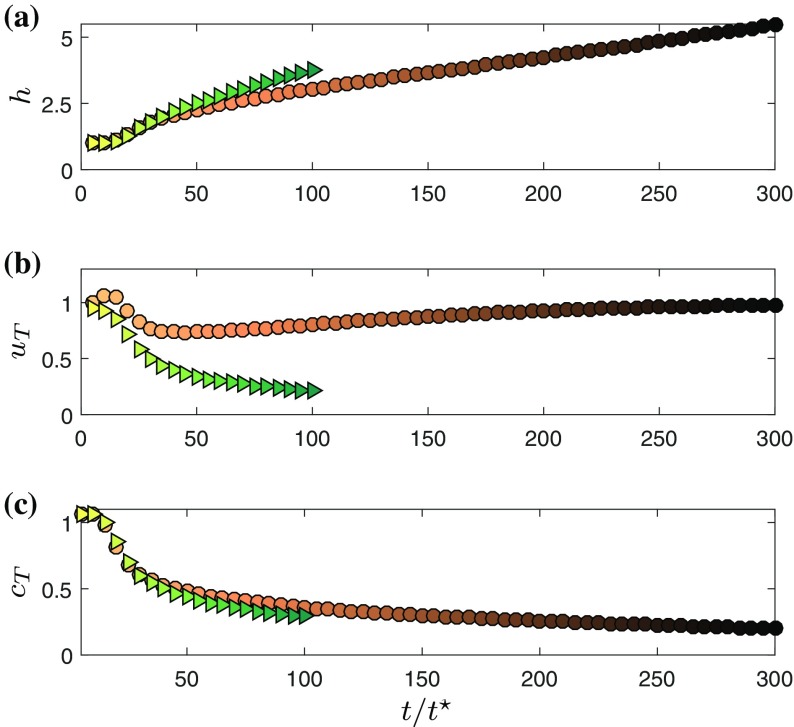
Temporal evolution of characteristic quantities for wall jet (WJ; *triangles*) and gravity current (GC; *circles*). **a** Thickness *h*. **b** Velocity $u_T$. **c** Scalar concentration $c_T$

The temporal evolution of the top-hat quantities in the simulations of the wall jet (WJ) and the gravity current (GC) are depicted in Fig. 2. For both flow types, the time is normalized by a reference time-scale based on initial conditions given by $t^{\star }\equiv h_0/U$. The early stages of both simulations are characterized by the transition to turbulent flow which happens rather quickly and is most notably observed in a steep drop of $u_T$ for $t<20t^{\star }$. If the influence of the wall is neglected, the self-similar inviscid scalings for WJ and GC are the same as the ones of temporal jets and plumes. For these cases it was found that $u_T\propto t^{-1/2}$, $h\propto t^{1/2}$ and $c_T\propto t^{-1/2}$ for jets [11] and $u_T\propto \text {const.}$, $h\propto t^{1}$ and $c_T\propto t^{-1}$ for plumes [20]. As will be discussed below, the WJ entrains more and hence the effective growth rate is larger. Consequently the simulation needs to be stopped earlier before the flow is influenced by the presence of the top-boundary of the numerical domain and the available simulation time is too short to determine the scaling consistently from the data (even though the trends are consistent). In contrast, the GC can be run for longer times without confinement effects and it is clear that $u_T$ tends towards a constant value (cf. Fig. 2b) and $h_T$ growths approximately linearly in time (see Fig. 2a).

**Fig. 3:**
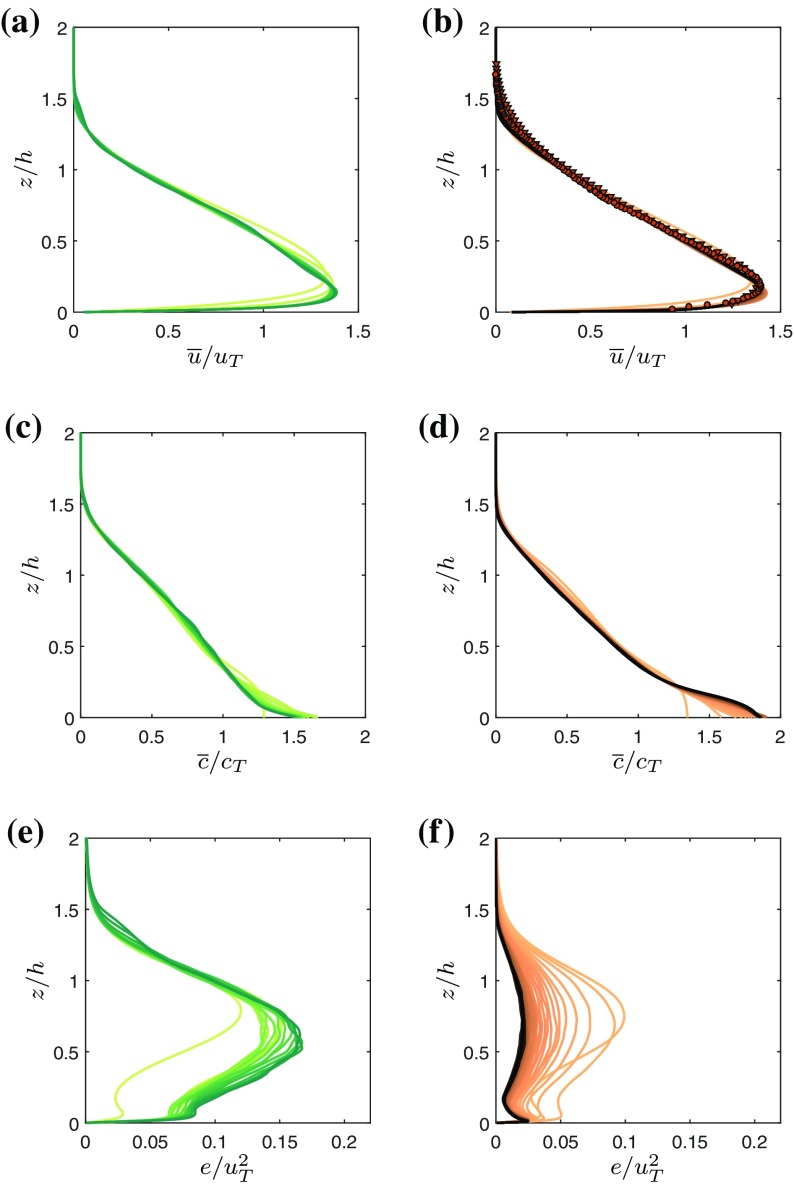
Self-similarity for WJ (**a**, **c**, **e**) and GC (**b**, **d**, **f**). **a**, **b** Mean streamwise velocity $\overline{u}$ (*lines*) along with experimental data from [6] (*symbols*) **c**, **d** scalar concentration $\overline{c}$. **e**, **f** Turbulence kinetic energy *e*. Different *shades of brown* indicate gravity current data at different times, *shades of green* represent wall-jet data. The *color-coding* for individual lines is the same as the one used for the *symbols* in Fig. 2 to convey the temporal correspondence

In order to asses the self-similarity of the flow we plot wall-normal profiles of $\overline{u}(t)$ and $\overline{c}(t)$ in Fig. 3 normalized by the respective top-hat values $u_T$ and $c_T$. In order to avoid clutter, the first three data points (which are roughly corresponding to the transitional flow stage) for each flow type were omitted. The different flow types and times are color coded using the same schemes as employed for the symbols in Fig. 2 in order to give an impression of the temporal evolution. Figure 3a, b convincingly show that the streamwise velocity distributions are self-similar to good approximation even shortly after transition to turbulence in both flows.

We find very good agreement of the DNS data with experimental data obtained by Krug et al. [6] (circles and triangles correspond to their ‘LD’ and ‘HD’ case, respectively). A distinct maximum of the velocity distribution is observed relatively close to the wall (at $z/h \approx 0.14$ followed by an approximately linear decrease of $\overline{u}$ reaching zero at $z/h \approx 1.5$ with little differences between the two flow types). In the near-wall region, i.e. below the approximate position of the velocity maximum, no collapse is expected as the inner region will be dominated by the classical inner scales $u_\tau$ (friction velocity) and $\nu /u_\tau$.

The collapse of the buoyancy profiles in the outer part of the flow in Fig. 3c, d is equally good as the one observed for $\overline{u}$. For the outer layer, it is remarkable how well the first order statistics agree between WJ and GC given the fact that the general flow evolution is completely different, as seen in Fig. 2.

From the plot of turbulence kinetic energy $e=0.5(\overline{{u}'^2}+\overline{{v}'^2}+\overline{{w}'^2})$ (Fig. 3e, f), it is evident that self-similarity is attained much later than for the first order statistics. Indeed, for the turbulent kinetic energy *e*, a full collapse of the profiles is only observed for $t/t^{\star }>50$ (WJ) and $t/t^{\star }>130$ (GC). When normalized by $u_T^2$, the turbulence levels in the WJ are observed to be considerably higher than those in the GC.

**Fig. 4:**
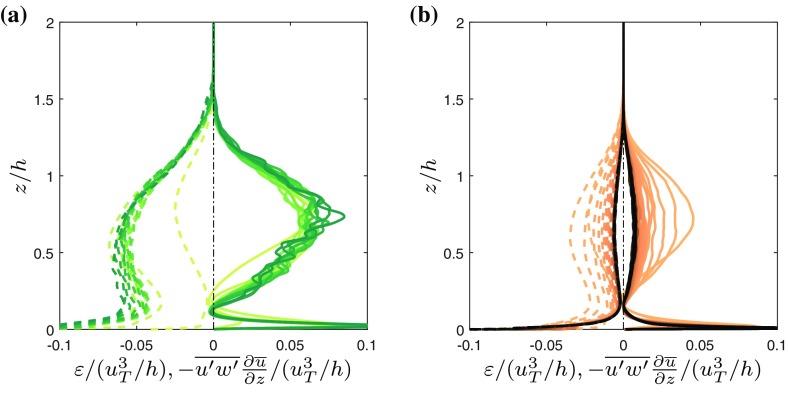
Mean rate of turbulent dissipation $\varepsilon$ (*dashed lines*) and shear production $-\overline{{u}'{w}'}\partial \overline{u}/\partial z$ (*solid lines*). **a** Wall-jet. **b** Gravity current. Different *shades of brown* indicate gravity current data at different times, *shades of green* represent wall-jet data. The *color-coding* is the same as the one used for the *symbols* in Fig. 2

The difference between the two flows is also clear from the shear production $-\overline{{u}'{w}'}\partial \overline{u}/\partial z$ of kinetic energy and the viscous dissipation of kinetic energy $\varepsilon = \nu \overline{(\partial u'_i /\partial x_j)^2}$, which are plotted in Fig. 4. Indeed, both terms are much larger in magnitude for the WJ than the GC case.

At the position of the velocity maximum the production term is 0 leading to a local minimum of *e* at the same position which appears more pronounced for the GC. This layer of low turbulence intensity inhibits mixing of the region close to the wall which again is reflected in higher values of $\overline{c}$ below $z/h=0.15$ and a strong negative gradient $\partial \overline{c}/\partial z$ in the proximity of the velocity maximum (cf. Fig. 3d). This effect is strengthened for the GC because negative $\partial \overline{c}/\partial z$ implies a stable stratification of the now active scalar which in turn inhibits mixing. The existence of an excess of negatively buoyant fluid near the wall was originally reported in [2]. Furthermore, note that the turbulence is in a local equilibrium between production and dissipation over the entire outer layer for the GC case, but not for the WJ case, where this only occurs in the top of the outer layer, say $z/h>1$.

## Turbulent entrainment

The rate at which the fluid layer grows due to turbulent entrainment is one of the most fundamental properties of these flows; it is usually quantified by the entrainment coefficient *E*, defined as

6 $$\begin{aligned} E = \frac{1}{u_T} \frac{ d h}{ d t}. \end{aligned}$$

For sufficiently high Reynolds and Péclet numbers, we expect that $E=f(\hbox {Ri})$, where

7 $$\begin{aligned} \hbox {Ri}= \frac{B_0 \cos \alpha }{u_T^2}. \end{aligned}$$

is the Richardson number of the flow. It is important to note that for the gravity current $\hbox {Ri}=\hbox {Ri}(t)$, as gravity performs work on the flow, thereby altering the flow statistics. Furthermore, note that the integral buoyancy *B* is conserved for these flows and thus $B=B_0$.

**Fig. 5:**
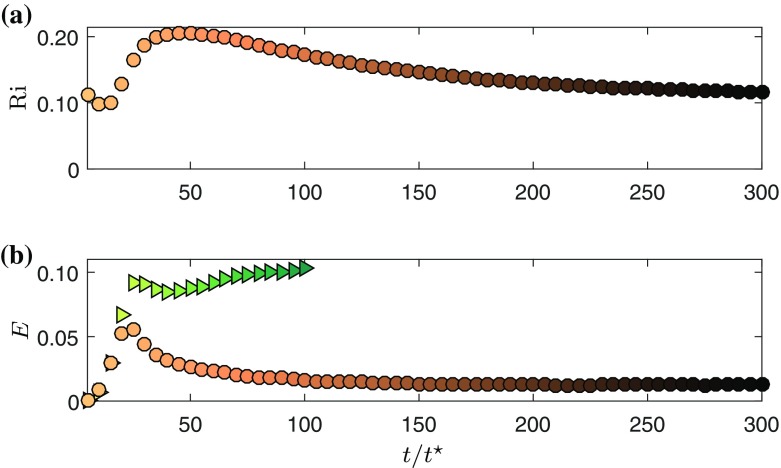
**a** Entrainment coefficient as a function of time. **b** Richardson number $\hbox {Ri}$ as a function of time. *Triangles* and *circles* represent wall-jet and gravity current data, respectively

For large *t*, the GC will attain a constant value of $\hbox {Ri}$. Here we note a correspondence with turbulent plumes, where a “pure” plume will have a constant value for $\hbox {Ri}$. If the flow has an excess (deficit) of momentum at the source the plume is referred to as forced (lazy) [21, 22]; the flow will adjust its own state due to the work done by gravity until it becomes pure. The evolution of $\hbox {Ri}$ for the gravity current in time (Fig. 5a), shows that $\hbox {Ri}$ initially falls and then recovers, reaching approximately a constant value at $t/t^*=200$.

The entrainment coefficient *E* is shown as a function of time in Fig. 5b. Clearly, *E* is much smaller in the GC than in the WJ. This effect is a consequence of the presence of a stable stratification in the GC and similar observations where made by many researchers dating back to the early work of [2]. This observation is consistent with the fact that the dimensionless turbulence production (Fig. 4), which has been shown to be closely linked to the entrainment coefficient [23] is much smaller for GC than for WJ.

**Fig. 6:**
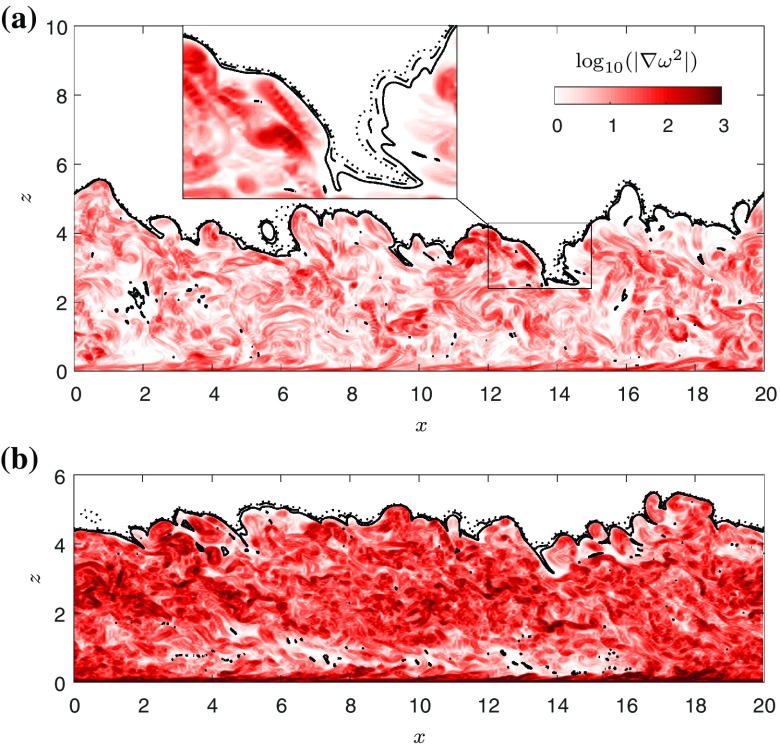
*Contour plot* of $\log _{10} | \nabla \omega ^2 |$, together with isolevels of $\omega _0^2 = 0.1\omega ^2_r$ (*dotted*), $\omega ^2_r$ (*dashed*) and $10\omega ^2_r$ (*solid*). **a** Wall jet at $t/t^{\star }=95$. **b** Gravity current at $t/t^{\star }=140$. The *inset* in **a** shows a blowup of the region marked with the *black triangle*

Since entrainment intrinsically is a multi-scale process, it is possible to link *E* to the small-scale entrainment occurring at the outer fringes of the turbulence, at the so-called turbulent-nonturbulent interface (TNTI) [12], which is the thin layer which links the irrotational ambient fluid to the turbulent interior of the flow. A suitable turbulence indicator is the enstrophy $\omega ^2 \equiv \omega _i \omega _i$, where $\omega _i$ is a component of vorticity. Figure 6 displays instantaneous contours of the gradient of the enstrophy fields in the WJ (Fig. 6a) and the GC (Fig. 6b). The times for the snapshots (and in the following) were chosen such that the top-hat width is approximately the same for both flow cases at $h=3.6$. Owing to the fact that the GC is continually driven by buoyancy while the WJ is lacking a source of energy, the levels of $\nabla \omega ^2$ are somewhat lower in the core of the WJ than in the GC. Also shown in Fig. 6 are three isocontours relating to threshold values of $\omega ^2/\omega ^2_r = 0.1$, 1 and 10 in both panels. $\omega ^2_r$ is a reference enstrophy threshold signifying the interface between the viscous superlayer and the buffer layer, see Fig. 8 for more details. At these low thresholds values, the contours are seen to trace the outer boundary of the flow faithfully. In view of the fact that the values of $\omega _0^2$ plotted cover two orders of magnitude, the variation of the interface position is only very small with no discernible differences between the two flows.

It is common to define a single threshold value $\omega _0^2$ and then test whether the local entrainment quantities are not overly dependent on its value [12]. This is not the approach adopted here; we follow [11] and choose a range of 33 threshold values $\omega _0^2$ that cover the interval $\omega _0^2 \in [10^{-8}, 10^0]$, which allows us to quantify the dependence on threshold value explicitly. The enstrophy threshold provides a single parameter to gauge the properties outside of the turbulent layer (for the smallest thresholds), the interfacial layer (the TNTI) and the core of the turbulence (for the high thresholds). In [11] it was shown that for a temporal jet four flow regions could be distinguished: the irrotational region, the viscous superlayer (VSL), the buffer region (BR) and the turbulent core (TC). Another notable fact from that paper was the formulation of a model which showed that the enstrophy is expected to drop of exponentially in the VSL. In order to avoid contamination from inner layer effects and focus on the TNTI dynamics exclusively, we exclude the region $z<0.5\,h_0$ from the calculations.

**Fig. 7:**
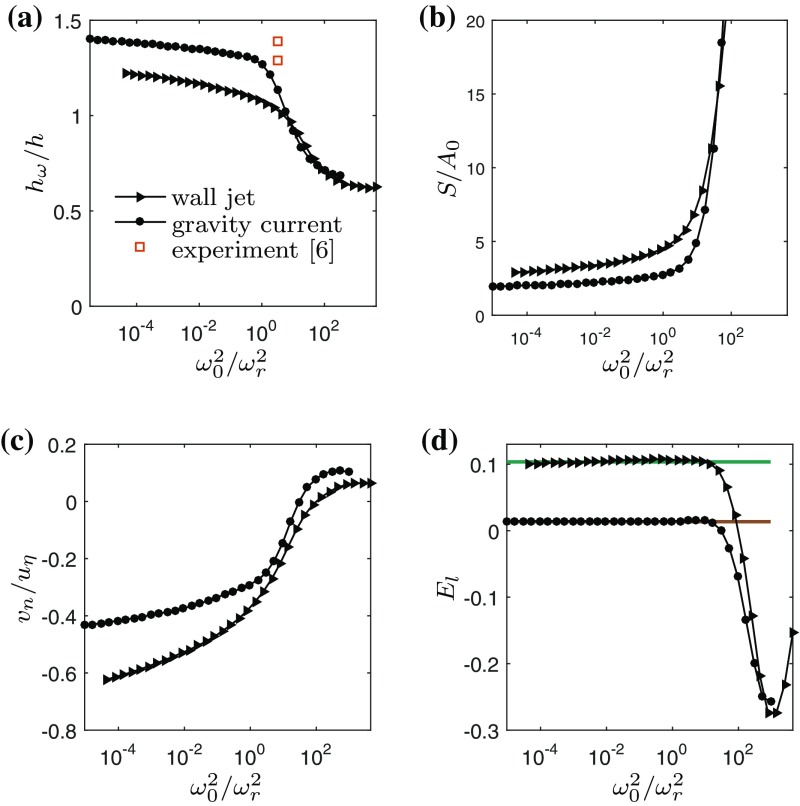
Small scale statistics of WJ (*triangles*) and GC (*circles*) for the instances shown in Fig. 6. **a** Ratio of mean interface position $h_{\omega }$ to top-hat width *h*. *Red symbols* show experimental results from Krug et al. [6]. **b** Ratio of surface area *S* to projected area $A_0 = L_x L_y$. **c** Local entrainment velocity $v_n$ normalised by $u_\eta$. **d** $E_l$ from interface based approach (*symbols*) compared to integral *E* (*horizontal lines*) at the corresponding times

Before quantifying small-scale entrainment properties of the iso-enstrophy surface, we focus on their geometric properties. In the remaining analysis we limit ourselves to the time-instances shown in Fig. 6. The dependence of the average *z*-position of the isosurface $h_\omega$ on the threshold value $\omega _0^2$, normalised by *h*, is shown in Fig. 7a. Here $\omega _r^2$ is a reference threshold that demarcates the end of the VSL and the beginning of the BR, which is defined as the location where 95 % of the interface propagation is due to viscous effects (see below).

For low $\omega ^2_0$ corresponding to the VSL there is only little variation and the values of $h/h_{\omega }$ are slightly larger than 1, consistent with the experimental results of Krug et al. [6]. The value quickly drops below 1 for thresholds values corresponding to the turbulent core. The DNS results confirm the general trend of increasing $h_{\omega }/h$ with increasing stratification that was pointed out in Krug et al. [6]. The relative location of the interface is consistent with the experiments in Krug et al. [6], as shown with the red squares. In the experiment, the relative viscous contribution to the outward propagation of the interface was $\approx$0.4–0.6. We plotted the points at the value for $\omega _0^2/\omega _r^2$ for which this was the case in the GC simulation.

The surface area *S* of the iso-enstrophy surface for $\omega _0^2$ is calculated in the DNS at runtime by counting the number of faces that form the interface between the regions for which $\omega ^2 > \omega _0^2$ and $\omega <\omega _0^2$. The surface area *S* is plotted in Fig. 7b as a function of $\omega _0^2$. The surface area in the VSL is only weakly dependent on the threshold value and its value remains larger than the projected area $A_0= L_x L_y$ because of the modulation by large scale structures in the flow. Furthermore, WJ has significantly more surface area than GC in the VSL. This is a direct effect of the buoyancy, which inhibits vertical motion. Inside the turbulence, the surface area increases rapidly with increasing $\omega _0^2$.

By examining the dynamics of iso-enstrophy surfaces, it is possible to determine a local entrainment velocity [10]

8 $$\begin{aligned} \hat{v}_{n}=-\frac{1}{|\varvec{\nabla }\omega ^2|} \frac{D\omega ^2}{D t}. \end{aligned}$$

Substituting the enstrophy balance equation

9 $$\begin{aligned} \frac{D}{D t} \left( \frac{\omega ^2}{2}\right) = \nu \varvec{\nabla }^2 \left( \frac{\omega ^2}{2}\right) + \omega _i \omega _j s_{ij} - \nu \varvec{\nabla }\omega _i \cdot \varvec{\nabla }\omega _i + \varvec{\omega } \cdot \varvec{\nabla }\times \varvec{b} \end{aligned}$$

into (8) and averaging over the enstrophy isosurface $\langle \cdot \rangle _S$, we obtain an expression for the average entrainment velocity $v_n$:

10 $$\begin{aligned} v_{n}\equiv \left\langle {\hat{v}_n}\right\rangle _S=v_n^{\mathcal {P}} + v_n^{\mathcal {D}} + v_n^{\mathcal {E}} + v_n^{\mathcal {B}}, \end{aligned}$$

where

$$\begin{aligned} v_n^{\mathcal {P}}&= -\left\langle {\frac{2\omega _i\omega _j s_{ij}}{|\varvec{\nabla }\omega ^2|}}\right\rangle _S,&v_n^{\mathcal {D}}&=-\left\langle {\frac{\nu \varvec{\nabla }^2\omega ^2}{|\varvec{\nabla }\omega ^2|}}\right\rangle _S, \\ v_n^{\mathcal {E}}&=\left\langle {\frac{2\nu \varvec{\nabla }\omega _i \cdot \varvec{\nabla }\omega _i}{|\varvec{\nabla }\omega ^2|}}\right\rangle _S,&v_n^{\mathcal {B}}&=-\left\langle {\frac{2\varvec{\omega } \cdot \varvec{\nabla }\times \varvec{b}}{|\varvec{\nabla }\omega ^2|}}\right\rangle _S. \end{aligned}$$

Here $v_n^{\mathcal {P}}$ represents interface motion due to vortex stretching, $v_n^{\mathcal {D}}$ due to enstrophy diffusion, $v_n^{\mathcal {E}}$ due to viscous dissipation of enstrophy and $v_n^{\mathcal {B}}$ represents interface propagation due to the baroclinic torque. Note that $v_n$ is positive along the gradient of enstrophy which points inside the turbulent region. Negative $v_n$ therefore corresponds to outward spreading of the interface.

Inside the viscous superlayer, the velocity scale is related to the Kolmogorov scale [10–12, 24]. This is evident in Fig. 7c, which displays $v_n$ normalised by the Kolmogorov velocity $u_\eta \equiv (\nu \varepsilon )^{1/4}$ where $\overline{\varepsilon }$ is the mean value for $0.45<z/h<0.55$ (cf. Fig. 4). However, $v_n$ is not entirely independent of $\omega _0^2$, with larger velocities observed for lower threshold values. As noted in [11], this is likely a low-Reynolds number effect due to the fact that $\eta /h$ is not infinitesimally small, as is corroborated by the fact that the threshold dependence is stronger for WJ than GC, the former being a problem in which the turbulence decays in time.

The relation between the integral and local entrainment can be quantified by turning to the entrainment volume flux $Q_e$. For the small-scale entrainment, $Q_e = - v_n S$, whilst for the large-scale entrainment, $Q_e = E u_T A_0 (h_\omega /h)$. Here, the factor $h/h_{\omega }$ accounts for the fact that due to self-similarity isosurfaces that lie further from (closer to) the wall as *h* need to propagate outwards faster (slower) than the top-hat width [11]. By equating these two expressions for $Q_e$, it is possible to infer *E* from the local framework according to

11 $$\begin{aligned} E_l = -\frac{v_n S}{u_T A_0}\frac{h}{h_{\omega }}. \end{aligned}$$

The results for $E_l$ at times corresponding to the snapshots in Fig. 6 are displayed in Fig. 7d (symbols) and compared to the corresponding values of *E* from Fig. 5b (solid coloured lines). We note excellent agreement for a wide range of $\omega ^2_0$ up to $\log _{10}\omega ^2_0/\omega _r^2 \approx 1$ for the GC and WJ.

Building on the convincing agreement between *E* and $E_l$ for a wide range of thresholds, we can scrutinise the entrainment process by studying the individual contributions to $v_n$ according to Eq. 10 in terms of the normalised contributions to the entrainment flux $Q_e = -v_n S$.

**Fig. 8:**
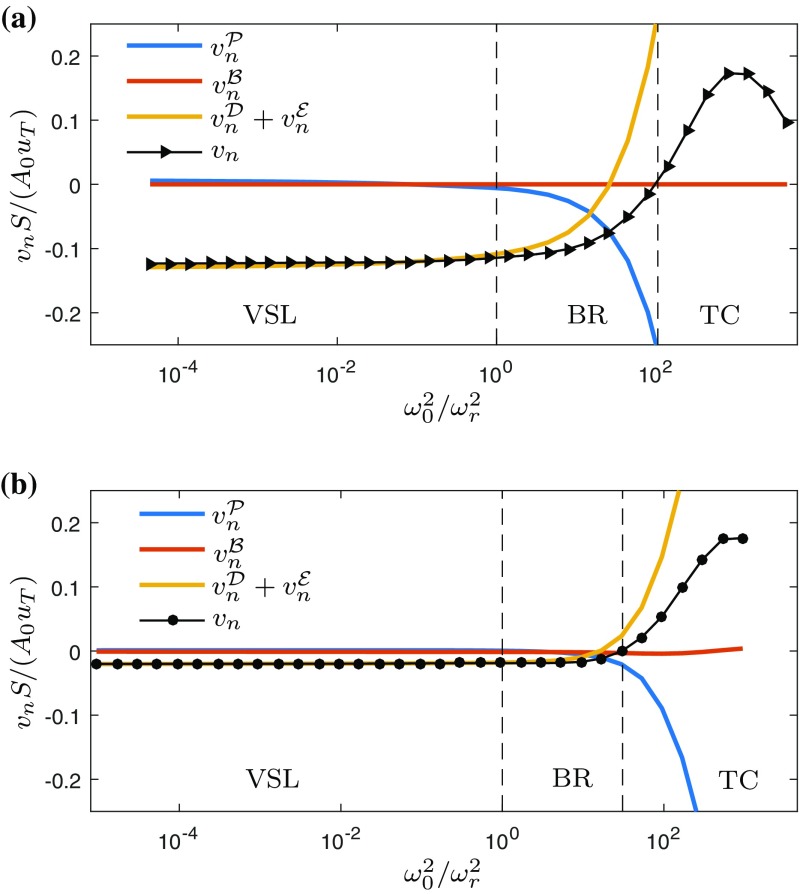
Normalised small-scale entrainment flux. **a** Wall jet. **b** Gravity current. *Vertical dashed lines* mark indicate the boundaries between the viscous sublayer (VSL), buffer region (BR) and turbulent core (TC), as defined by [11]

Figure 8 shows the volume flux through the enstrophy isosurface due to different components of $v_n$ for a wide range of threshold values for WJ (Fig. 8a) and GC (Fig. 8b). We find that for low $\omega _0^2$ entrainment is almost entirely a viscous process as conjectured by [25] and confirmed by e.g. [6, 10, 11, 26, 27]. This is evident from the fact that for low thresholds the sum of the viscous contributions $v_n^{\mathcal {D}}+v_n^{\mathcal {E}}$ and the net $v_n$ lie right on top of each other while the other components are close to zero. As mentioned before, the VSL is assumed to start where $(v_n^{\mathcal {D}}+v_n^{\mathcal {E}})/v_n>0.95$. The VSL can be seen to extend over four orders of magnitude in $\omega _0^2/\omega _r^2$, which corresponds to approximately $2 \eta$ in space [11]. It is only for higher $\omega _0^2$ in the buffer region (BR), which extends from the edge of the VSL to the threshold for which $v_n =0$, that the vortex-stretching term $v_n^{\mathcal {P}}$ plays a significant role. At the highest $\omega _0^2$, $v_n$ is positive i.e. the interface is receding which characterizes the turbulent core (TC) according to the definition of [11].

Given the fact that the baroclinic torque contribution $v_n^{\mathcal {B}}$ is a direct consequence of the presence of buoyancy, it might seem likely to assume that it is responsible for reducing the entrainment rate in the presence of a stable stratification. However, as can be seen from Fig. 8b, the contribution of the baroclinic torque (red line) is small throughout the full range of thresholds investigated here. This observation confirms the experimental finding of Krug et al. [6] who also found that the $v_n^{\mathcal {B}}$ is negligible. Further, consistent with the experimental results is the fact that $v_n^{\mathcal {B}}$ (albeit small compared to the other contributions) attains negative values, i.e. it enhances the outward spreading of the interface (Fig. 8b). This implies that in gravity currents the ‘plume-like’ or ‘unstable’ configuration prevails for the baroclinic term. As discussed in [20], in this case the wall normal gradient of buoyancy along with the streamwise component of the gravitational acceleration leads to a vorticity component that is aligned with that of the mean shear such that the baroclinic enstrophy production is positive (positive $v_n^{\mathcal {B}}$). In contrast, when the buoyancy gradient and the gravitational acceleration are anti-aligned (‘stable’ configuration), distortions of the stably stratified buoyancy field by eddies lead to a restoring torque which results in negative baroclinic production (positive $v_n^{\mathcal {B}}$).

Finally, we determine the cause of the reduction in the entrainment coefficient. The individual terms comprising *E*_*l*_ (Eq. 11), namely* v*_*n*_/*u*_*T*_,* S*/*A*_*0*_ and $h/h_{\omega}$, were calculated and then averaged over a range of thresholds $10^{-3}< {\omega}^{2}_{0} / {\omega}^{2}_{r}< 10^{-1}$. The individual terms only vary marginally over this region (Fig. 7), and the results are presented in Table 2. The final row of the table contains the ratio of the GC to the WJ case, and clearly shows that at the present value of Ri the main reason for the reduction in* E*_*l*_ is the reduction in * v*_*n*_/*u*_*T*_, followed by the surface area* S*/*A*_0_. The factor $h/h_{\omega}$ only marginally influences the reduction in* E*_*l*_. The strong reduction in * v*_*n*_/*u*_*T*_ is interesting, particularly in the light of Fig. 7c, which shows consistent with [6] that the ratio * v*_*n*_/*u*_*T*_ is ~0.5 in both cases. Clearly, the stable stratification suppresses the (normalised) turbulence production and dissipation (cf Fig. 4), resulting in lower values of $u_{\eta}$ and thus* v*_*n*_. Furthermore, the surface area is reduced by over 30% in the GC case, which is the second major effect of the stable stratification created by the GC. Both the reduction in * v*_*n*_/*u*_*T*_ and* S*/*A*_*0*_ and can be expected to have a dependence on Ri, which will be considered in in future work.

**Table 2:** Magnitude of the terms comprising* E*_*l*_ (Eq. 11) for the WJ and GC cases

	* v*_*n*_/*u*_*T*_	$S/A_0$	$h/h_\omega$	$E_l$
WJ	0.036	3.429	0.861	0.105
GC	0.008	2.314	0.743	0.015
GC/WJ	0.237	0.675	0.863	0.138

The product of the first three columns produces* E*_*l*_ displayed in the last column. The bottom row contains the ratio of the two rows above

*WJ* wall-jet simulation, *GC* gravity current simulation

## Conclusions

In this paper, a temporal version of the classical Ellison and Turner gravity current experiments was introduced. Results from direct simulation of a temporal wall jet and a gravity current were performed with the aim of identifying the effects of buoyancy on small-scale entrainment. We find that a larger entrainment coefficient for the wall jet compared to the gravity current can be attributed to stronger turbulence production in the former. It was shown that the integral entrainment coefficient *E* can be related to the small-scale entrainment over a large range of enstrophy thresholds. We find in both cases a viscous superlayer at the outer fringes of the turbulent flow region where the local entrainment velocity is dominated by viscous diffusion of enstrophy. In both cases the entrainment scales with the Kolmogorov velocity in the viscous superlayer. The decomposed local entrainment flux indicates that, consistent with [6, 14], the baroclinic torque term is negligible. Further work is needed in analysing the effects of buoyancy on the area and shape of enstrophy isosurfaces.

Overall, the picture that emerges is that the reduction in the entrainment coefficient due to stratification is caused by: 1) the reduction of* v*_*n*_/*u*_*T*_; and 2) the reduction of the surface area of the interface. The ultimate aim of our work is to provide the observed entrainment relations $E(\hbox {Ri})$ with firm physical foundations. These foundations may help in reducing the enormous scatter in observations for inclined gravity currents, for example for the case of oceanic overflows [4].
